# Panning of a Phage Display Library against a Synthetic Capsule for Peptide Ligands That Bind to the Native Capsule of *Bacillus anthracis*

**DOI:** 10.1371/journal.pone.0045472

**Published:** 2012-09-19

**Authors:** Michael Beer, Chun-Qiang Liu

**Affiliations:** Human Protection and Performance Division, Defence Science and Technology Organisation, Fishermans Bend, Victoria, Australia; University of Iowa Carver College of Medicine, United States of America

## Abstract

*Bacillus anthracis* is the causative agent of anthrax with the ability to not only produce a tripartite toxin, but also an enveloping capsule comprised primarily of γ-D-glutamic acid residues. The purpose of this study was to isolate peptide ligands capable of binding to the native capsule of *B. anthracis* from a commercial phage display peptide library using a synthetic form of the capsule consisting of 12 γ-D-glutamic acid residues. Following four rounds of selection, 80 clones were selected randomly and analysed by DNA sequencing. Four clones, each containing a unique consensus sequence, were identified by sequence alignment analysis. Phage particles were prepared and their derived 12-mer peptides were also chemically synthesized and conjugated to BSA. Both the phage particles and free peptide-BSA conjugates were evaluated by ELISA for binding to encapsulated cells of *B. anthracis* as well as a *B. anthracis* capsule extract. All the phage particles tested except one were able to bind to both the encapsulated cells and the capsule extract. However, the peptide-BSA conjugates could only bind to the encapsulated cells. One of the peptide-BSA conjugates, with the sequence DSSRIPMQWHPQ (termed G1), was fluorescently labelled and its binding to the encapsulated cells was further confirmed by confocal microscopy. The results demonstrated that the synthetic capsule was effective in isolating phage-displayed peptides with binding affinity for the native capsule of *B. anthracis*.

## Introduction

*Bacillus anthracis* is capable of infecting many mammal species (including humans), and the acute form of infection, commonly referred to anthrax, is often fatal [1]. Infections arise from the highly durable and dormant form of *B. anthracis* known as endospores, which are formed from the metabolically active vegetative form in response to nutrient deficiency and/or environmental stress. Entry of endospores into an animal host can occur through cutaneous contact, ingestion or inhalation; the latter two routes of infection having the greatest chance of developing systemic infection and therefore highest mortality. Endospores germinate into metabolically active vegetative bacilli after being enveloped by macrophages and transported to lymph nodes, where they rapidly multiply. Vegetative bacilli are then released into the bloodstream, where they produce anthrax toxin, which is comprised of three proteins known as edema factor (EF), lethal factor (LF), and protective antigen (PA), encoded by the pXO1 plasmid. After binding to cell surface receptors, PA undergoes protease cleavage which then enables it to form a heptamer complex. This complex, known as a prepore, can bind both EF and LF. The tripartite toxin enters the host cell *via* endocytosis, resulting in edema (EF) and kinase inactivation (LF) [1].

The ability of *B. anthracis* to generate a capsule is also an essential component of its capacity to induce anthrax [2], [3]. The capsule of *B. anthracis* is comprised almost exclusively of a polymer of γ-D-glutamic acid residues (γPGA), which is resistant to protease activity [4]. The number of glutamic acid residues ranges from 1000 to 2500 [4], and the genes for capsule synthesis located on the pXO2 plasmid have also been well characterised [5]. γPGA is negatively charged, which enables the vegetative cells to evade phagosytosis by macrophages [6]. Given its crucial role in virulence, γPGA has been targeted for both active and passive immunization against anthrax infection [7], [8], [9]. Further, γPGA may also serve as a biomarker for early diagnosis of anthrax infection, because measurable levels of γPGA were produced as early as 24 hours after infection in a mouse model [7].

Several monoclonal antibodies have been produced for use in passive immunotherapy against anthrax [7], [8], [9] and immunoassays for γPGA in early diagnosis of anthrax [7], [10]. However, due to the poor immunogenicity, the capsule had to be pre-conjugated to either an immunogenic protein carrier [9], [11], [12], [13] or CD40 mAb [7], [8]. Most of the antibodies showed varying levels of protection when tested in a murine model of pulmonary anthrax [8]. Clearly, there is a need for alternative types of binding molecules that target the capsule of *B. anthracis*.

Phage display technology has proven highly effective in discovering peptides (including antibody fragments) with affinities to virtually any target. As drug candidates, peptides are often preferred to antibodies because of their lower manufacturing costs, greater stability and better organ or tumour penetration [14]. To date, several oligopeptides [15] and human antibody-like fragments have been isolated from phage display libraries and shown to have high affinity for *B. anthracis* endospores [16], [17], LF [18], and PA [19], or multiple/associated toxin components [20], [21], [22].

Here, we report for the first time the isolation and initial characterisation of phage-displayed peptides that bind to the capsule of vegetative cells of *B. anthracis*. This work represents a first step towards the development of novel therapeutic agents that allow selective killing of *B. anthracis* cells.

## Methods

### Phage Display Library and Panning Procedure

The Ph.D.-12™ phage display peptide library was purchased from New England Biolabs (Ipswich, MA, USA). The library displays random 12-mer peptides fused to the minor coat protein (pIII) of M13 phage. The following oligo-peptides were synthesized by AnaSpec (CA, USA): CL-1 (Biotin-LC-(γ-D-Glu)_12_-NH_2_), CL-2 (Biotin-LC-Gly-Gly-Pro-His-Ser-Gly- NH_2_), CL-3 (BSA-Cys-(γ-D-Glu)_12_-NH_2_), CL-4 (Acetyl-(γ-D-Glu)_12_- NH_2_) and CL-5 (Acetyl-Leu-Gly-Thr-Pro-His-Ser-Gly-Thr-Arg-Leu-Ser-Glu-NH_2_). LC is a 6-aminohexanoic acid spacer molecule used to minimize potential steric hindrance effects on the peptide. CL-1 and CL-3 are synthetic capsules, each consisting of 12 D-glutamic acid residues linked to biotin and BSA, respectively. CL-4 is a non-biotinylated version of CL-1. CL-2 and CL-5 are non-capsule control peptides.

Panning of the phage display library was carried out as per manufacturer’s instructions with modifications. Briefly, the synthetic capsule peptide CL-1 was captured via strepavidin conjugated magnetic beads and the non-biotinylated control peptide CL-5 was added in excess at the beginning of the each cycle to help eliminate non-specific binders. The screening stringency was increased by decreasing the concentration of the coated target peptide CL-1 and increasing the detergent percentage in the washing buffer. From the 1^st^ to the 3^rd^ round of selection, acidic elution was carried out to collect more binders. However in the 4^th^ round, competitive elution with a high concentration of CL-4 (the non-biotinylated target peptide) was performed to enrich specific binders. A 10-fold serial dilution of the phage particles from the 4^th^ round elute was prepared in Luria Broth, and added to a freshly prepared culture of *E. coli* ER2738 (New England Biolabs). The culture was incubated at room temperature for 10 minutes before plating out. Random single plaques were inoculated into *E. coli* ER2738 and incubated at 37°C for 5 hours.

### PCR and Nucleotide Sequencing

The DNA sequence of the 12-mer peptide in each phage clone was amplified by PCR using primers M13KE-f1 (5′-GTTGTTGTCATTGTCGGCGCA-3′) and M13KE-r2 (5′-GCCCTCATAGTTAGCGTAACG-3′). The temperature cycling consisted of an initial step of 5 minutes at 94°C, followed by 30 cycles of 96°C for 10 seconds, 50°C for 5 seconds, and 60°C for 4 minutes, and then held at 4°C. The resulting PCR product was purified with QIAquick PCR Purification Kit (Qiagen GmbH, Hilden Germany) and quantified with a NanoDrop spectrophotometer (Thermo Scientific, Wilmington, DE, USA). The DNA sequencing reactions were prepared using BigDye Terminator Ready Mix v3.1 (Roche), and sequencing reactions were analysed on an ABI PRISM 3100 Genetic Analyzer (Applied Biosystems, Mulgrave VIC, AUSTRALIA).

### Extraction of Capsular Material

The capsular material was extracted from ΔAmes-1, a non-toxigenic, encapsulated strain of *B. anthracis* which harbours the pXO2 plasmid responsible for capsule synthesis, but lacks the pOX1 plasmid encoding the three toxin components (Welkos, Little et al. 2001). The ΔAmes-1 strain was kindly provided by Dr J. Bates (Queensland Health Forensic and Scientific Services, Australia). A nutrient agar plate (3 g/L beef extract, 5 g/L peptone and 15 g/L agar) containing 0.8% NaHCO_3_ (added as an 8% (w/v) solution to molten agar at 50°C) was inoculated with *B. anthracis* ΔAmes-1, and incubated overnight at 37°C. A single colony from the nutrient agar plate was used to inoculate 4 mL of nutrient broth containing 0.8% (w/v) NaHCO_3_ in sterile 50 mL tube. The tube was shaken at 175 rpm for 24 hours at 37°C. The culture was spread onto 10 nutrient agar plates (50 µL each), and the plates incubated in an anaerobic jar containing about 15% CO_2_ that was generated by a Gas Generating Pouch (Oxoid, Melbourne, Australia). Plates were incubated for 48 hours at 37°C, after which growth was harvested with a cell scraper (Sigma, Melbourne, Australia) and the bacteria resuspended in 25 mL 1× PBS (8 g/L NaCl, 0.2 g/L KCl, 1.44 g/L Na a_2_HPO_4_, 0.24 g KH_2_PO_4_, pH 7.4) containing 4% (v/v) formaldehyde (Sigma), and incubated for 48 hours at 37°C. Two and a half grams of solid sodium acetate (Sigma) was added to the remaining suspension and agitated until dissolved. Glacial acetic acid (250 µL) (Sigma) was then added and the suspension mixed thoroughly. Cells were pelleted by centrifugation at 2 500× g for 5 minutes and the supernatant filtered through a 0.2 µm filter (MilliPore, North Ryde NSW, Australia). Two volumes of absolute ethanol were added to the filtrate, and mixed thoroughly. Capsular material was left to precipitate for 30 minutes at room temperature, before being collected by centrifugation at 5 500× g for 15 minutes at room temperature. The supernatant was discarded and the precipitate resuspended in 1.0 mL of sterile MilliQ water. The amount of capsular material was quantified with a NanoDrop Spectrophotometer (ThermoFisher Scientific).

**Table 1:** Amino acid sequences of the 12-mer peptides carried by selected phage clones.

Phage clone^a^	Translated amino acid sequence^b^	Peptide name^c^
P01(26/72)	DSS*R* **IPM**Q**W** *H* **P**Q	G1
P04(15/72)	**Y**TMQ**Y**S**LI**N*H* **P**Q	G2
P26(3/72)	S*H*S**AMY** E **Y** D **A**ST	G3
P50(3/72)	*H*S*H* **P**S*K* **FPALAY**	G4

a The designation of each phage clone is given along with their representation within the sequenced clones shown in parentheses.

b The translated amino acid sequence, determined from the Ph.D-12 insert library sequence, is also given (underlined - acidic residues, *italics* - basic residues, **bold** - neutral hydrophobic residues, normal – neutral hydrophilic residues).

c Peptide name designates the representative synthesised peptide.

### Enzyme-linked Immunosorbent Assay (ELISA)

Formaldehyde treated *B. anthracis* ΔAmes-1 cells were diluted to an OD_600_ of 0.5 in coating buffer (0.05 M NaHCO_3_ pH 9.6), and extracted capsular material diluted to 1 µg/mL in coating buffer. They were then added separately to wells (100 µL each) of a Nunc MaxiSorp F96 microplate (ThermoFisher Scientific), the plate then incubated overnight at 4°C. Wells were washed 3 times with PBST, and blocked for 1 hour at room temperature with PBST (PBS buffer with 0.2% v/v Tween20) containing 1.5%w/v BSA. Phage preparation (1.5×10^12^ pfu/mL) was diluted 1∶10 in PBST, added to designated wells (100 µL per well), incubated for 1 hour at room temperature, and washed three times in PBST. Mouse anti-M13 bacteriophage antibody (Sigma) was diluted 1∶2000 in PBST and added at 100 µL to each well. The plate was incubated for 1 hour at room temperature, and then washed three times in PBST. To each well, 100 µL of a 1∶2000 dilution of Anti-mouse IgG conjugated to Alkaline Phosphatase (Sigma) in PBST were added, incubated for 1 hour at room temperature, and washed three times in PBST. To each well, 100 µL of 1 mg/mL of 4-nitrophenyl phosphate disodium salt hexahydrate in 0.2 M Tris buffer (Sigma) was added. The plate was protected from light and shaken at room temperature for 1 hour. Absorbance was read at a wavelength of 405 nm with a Synergy HT Multi-Mode Microplate Reader (Bio-Tek, Winooski, VT, United States).

**Figure 1:**
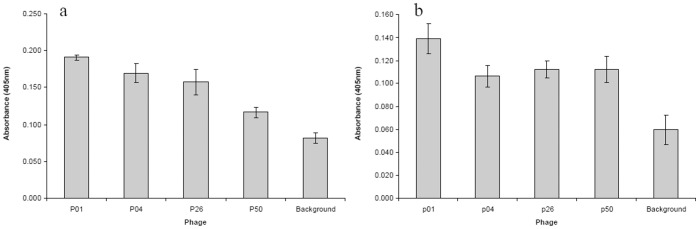
Binding assays for phage particles. Affinity between phage particles with encapsulated *B. anthracis* cells(a) and extracted capsule (b) was assessed by ELISA. The error bars indicate a confidence interval of 95%.

The synthetic peptides were manufactured and conjugated to BSA by ANASpec. The conjugated peptides were each diluted to 1.0 µg/mL in coating buffer (0.05 M NaHCO_3_ pH 9.6), and 100 µL added to a Nunc MaxiSorp F96 microplate (ThermoFisher Scientific) in triplicate for each target, and incubated at 4°C overnight. Plates were washed three times in PBST (PBS buffer with 0.2% v/v Tween20), after which 350 µL of BSA-PBST (0.25%w/v BSA in PBST) was added to each well and incubated for 1 hour at room temperature with gentle shaking (about 50 rpm). Each well was washed three times with 350 µL of PBST. Washed formaldehyde fixed *B. anthracis* ΔAmes-1 cells were diluted to an OD_600_ of 0.5 in BSA-PBST, and extracted capsular material diluted to 1.0 µg/mL in BSA-PBST. 100 µL of these preparations were added separately to triplicate wells coated with each peptide. The plate was incubated at room temperature for 1 hour with gentle shaking and washed three times in PBST. Monoclonal mouse anti-spore antigen (Hytest, Turku Finland) was added to the wells to which encapsulated cells were bound, and 100 µL of a single domain fragment expression culture lysate (diluted 1∶10 in PBST) known to bind to capsule extract (unpublished) was added to the wells to which extract was bound. The plate was incubated at room temperature for 1 hour with gentle shaking and washed three times in PBST. 100 µL of a 1∶2000 dilution of goat anti-mouse antibody conjugated to alkaline phosphatase (Sigma) in PBST were added to each well to which BA cells were previously bound, and 100 µL of a 1∶2000 dilution of Rabbit Anti-E tag Polyclonal Antibody (Thermo) in PBST were added to each well to which capsule extract was previously bound. The plate was incubated for 1 hour at room temperature, and then washed three times in PBST. 100 µL of a 1∶2000 dilution of anti-Rabbit IgG conjugated to Alkaline Phosphatase (Sigma) in PBST were added to each well to which capsule extract was previously bound and incubated for 1 hour at room temperature, and washed three times in PBST. After each well was washed once with 100 µL of distilled water, 100 µL of 1 mg/mL of 4-nitrophenyl phosphate disodium salt hexahydrate in 0.2 M Tris buffer (Sigma) was added to each well, and the plate was shaken at room temperature for 1 hour. Absorbance was read at 405 nm in a Synergy HT Multi-Mode microplate reader (Bio-Tek). All appropriate controls were carried out during the ELISA experiment, including a background control from which the primary binding (encapsulated cells/capsule extract) was omitted.

**Figure 2:**
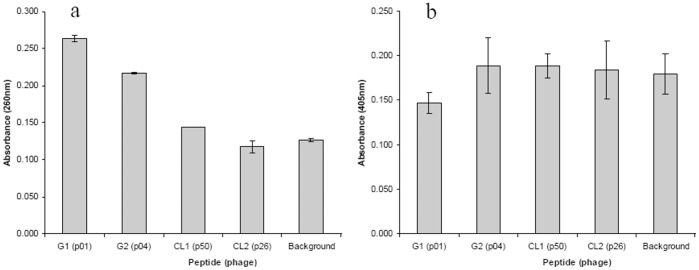
Binding assays for peptides detached from phage particles. Affinity was assayed by ELISA for the synthetic peptides derived from the variable region of phage clones with encapsulated *B. anthracis* cells (a) and extracted capsule (b). The error bars indicate a confidence interval of 95%.

### Confocal Microscopy

Peptide-BSA conjugates were labelled with AlexaFluor488 using the AlexaFluor488 Microscale Labelling Kit (Molecular Probes), as per the manufacturers instruction. BSA conjugated forms of the peptides G1 and CL3 (AnaSpecUSA) were diluted to 1.0 mg/mL in sterile 1×PBS, and the dye added at a ratio of 1∶30 (protein: AlexaFluor288). Absorbances at 280 nm and 494 nm were determined using the NanoDrop Spectrophotometer (Thermo), and the number of dye molecules per protein molecule was approximately 2.2 and 1.8 for G1-BSA and CL3-BSA respectively. Two *B. anthracis* strains were used: ΔAmes-1 and Sterne 34F2. The latter is a vaccine strain that is devoid of the capsule plasmid pXO2 (Green, Battisti et al. 1985). The two strains were cultured in nutrient media for 24 h at 37°C, and treated with 4% formaldehyde as detailed previously. Cells for each culture were collected by centrifugation (3500× g for 5 mins), and washed three times in 1× PBS. Cell suspensions were adjusted to an OD_600_ of 0.40, and diluted 1∶10 with 1× PBS. The suspended cells (20 µL) were spotted onto a glass cover slip, and allowed to dry at 20°C for 20 minutes. Slides were blocked by immersion into PBTS solution containing 0.25% (w/v) BSA for 30 minutes at room temperature. Cover slips were washed with 1× PBS, and stained with 50 µL of a 0.8 mg/mL solution containing one of the Alexifluor488 labelled peptides (G1-BSA conjugate or CL3-BSA conjugate). After 5 minutes the cover slips were washed twice in 1×PBS. Fluorescence confocal microscopy was performed using a Leica TCS SP5 spectral confocal microscope based on a DMI600 inverted microscope. A 63× oil immersion lens with a numerical aperture of 1.4 was used for all images. A 488 nm line from an Ar laser was used for excitation of AlexaFluor488, and emission detected with a standard fluorescein filter (522±35 nm). Image manipulations of confocal microscope data were performed using Leica Applications Suite, and were limited to brightness, contrast, intensity and tone curve adjustments.

**Figure 3:**
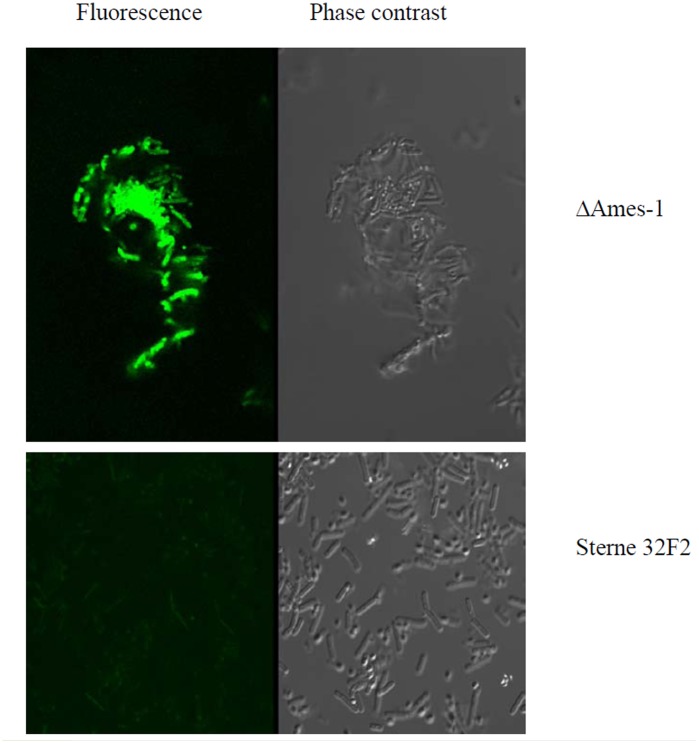
Confocal microscopy and image analysis. Binding of peptide G1 to *B. anthracis* encapsulated cells was determined by confocal laser scanning microscopy and digital image analysis showing selective binding of AlexaFluo488 labelled peptide G1 to encapsulated cells of *B. anthracis* (ΔAmes-1) over unencapsulated cells of *B. anthracis* (Sterne 32F2). Scale bar = 5 µm.

## Results

### Panning of Phage Display Peptide Library Ph.D.-12

Following four rounds of panning, 80 phage clones were randomly selected and the region flanking the DNA insert in the gene III of each clone was amplified using primers M13KE-f1 and M13KE-r2. No PCR product was obtained on eight clones. Of the remaining 72 clones sequenced, clone p01 was found to be the most represented, comprising 36% of all the sequenced clones. Another clone which had a similar motif to p01 (three identical terminal residues) and representation in both screenings (about 21%) was p04. Phage clones p26 and p50 were represented three times and also warranted further investigation. The remaining clones had variable sequences which were only represented once, and were thus considered to have a lesser γPGA binding potential. The amino acid sequence of the peptides p01, p04, p26 and p50 and their representation among the sequenced clones, are given in Table 1.

### Binding of Phage Particles to *B. anthracis* Capsule Extract and Encapsulated Cells

The capsule was extracted from the vegetative cells of *B. anthracis* ΔAmes-1 as described in the Materials and Methods. A sample of the capsule extract was analysed on an SDS-PAGE gel. A high molecular weight band (greater than 250 kDa) stained positive with methylene blue, but negative for coomassie blue indicating the presence of γPGA (data not shown). Phage particles were prepared from the clones listed in Table 1 and assayed by ELISA for binding to the capsule and the vegetative cells of *B. anthracis*, respectively. Phage particles were first adsorbed to immobilised encapsulated cells or capsule extract, and then detected with an antibody to M13 bacteriophage. All the phage particles except p50, produced comparable signals against encapsulated cells (Fig. 1a). Clone p01 resulted in the greatest signal against the capsule extract, while the other clones gave a lower but comparable result to one another (Fig. 1b).

### Binding of Phage Particle-free Peptides to *B. anthracis* Capsule Extract and Encapsulated Cells

Five peptides were synthesised, four of which were based on the sequence information in Table 1, and the fifth, named G5 (SDYPWKNYHEAQ), was from the insert of a random phage clone that did not bind to the capsule of *B. anthracis*. A short spacer (GGGC) was also added at the 3′ end of each peptide. The five synthetic peptides were each conjugated *via* the cysteine residue to BSA. The binding of BSA-peptide conjugate to synthetic PGA was confirmed by ELISA, in which biotinylated-PGA was adsorbed to plate bound peptides and the former then detected with streptavidin-AP conjugate (data not shown). Encapsulated cells or capsule extract was adsorbed to plate bound peptides, the former then detected with an antibody generated to target capsule (unpublished). The ELISA results for binding to encapsulated cells are shown in Fig. 2a. G1, the peptide derived from phage clone p01, had the greatest ELISA signal, with reduced signals obtained for peptides G2 (p04) and G4 (p50). The random peptide G5 did not provide a signal significantly greater then the background control. However, no binding was detected between the capsule extract and synthetic peptides (Fig, 2b), as all peptide signals were indistinguishable from the background control. It is worth noting that these ELISA data (Fig. 1 and 2, a and b) are not quantitatively comparable to one another as no other no characterised antibody which targets capsule was readily available to us.

Surface Plasmon Resonance (SPR) was employed to examine the binding between the G1 peptide and extracted capsule, but experiments were not successful (data not shown). As a consequence, confocal microscopy was carried out to visualise of the binding to encapsulated cells, as detailed below.

### Confirmation of Peptide Binding to *B. anthracis* Encapsulated Cells by Confocal Microscopy

Peptide G1 was fluorescently labelled with AlexaFluo488 and then mixed with encapsulated *B*. *anthracis* (ΔAmes-1) cells and un-encapsulated *B. anthracis* (Sterne 32F2) cells. Although cells of both strains exhibited auto-fluorescence, the signal obtained with the encapsulated strain was readily discernible from that of the un-encapsulated strain (Fig. 3). In another experiment, the two strains were mixed, separately, with the control peptide G5 labelled with AlexaFluo488, however no signal was detectable by confocal microscopy (data not shown). The results clearly demonstrated that the G1 peptide was able to bind to the native capsule of vegetative cells of *B. anthracis*.

## Discussion

In this work, we used a synthetic capsule peptide of 12 γ-D-glutamic acid residues to pan a phage display library for peptide ligands that bound directly to the native capsule of *B. anthracis*. Even though the phage display library could have been panned against vegetative cells of *B. anthracis* or its capsule extract, the synthetic peptide was chosen for the following reasons. First, neither encapsulated cells nor its capsule extract was free from non-γPGA antigens, which could result in the selection of phage particles with an affinity for the non-γPGA antigens. In contrast, the synthetic peptide could be readily synthesized and purified. Second, the preparation of encapsulated cells or its capsule extract involves handling of pathogenic *B. anthracis*, with stringent requirements for biosafety and physical containment. Finally and most importantly, similar oligo-γPGA-peptides (5, 10, 15 and 20 monomers) had been used successfully to raise antibodies with affinity for the native capsule in animals including guinea pigs, rabbits and mice [9], [12].

A peptide with high affinity to the capsule of *B. anthracis* may be used potentially to deliver a therapeutic agent to the surface of the vegetative cells of *B. anthracis*. Examples of such agents include those that degrade capsular material, such as capsule depolymerase [3], [23], immunostimulatory components, and bioreactive peptides that reduce microbial function, such as antimicrobial peptides (AMPs) [24], [25]. Over the last few years, we and others have identified a number of AMPs that are potent against *B. anthracis* [26]. Targeting of such AMPs, through conjugation with a capsule-binding peptide, to the vegetative cells of *B. anthracis* would be highly beneficial, as most AMPs discovered to date have a broad range of activity killing both pathogenic and beneficial bacteria. As demonstrated for *Pseudomonas mendocina* [27], the presence of such a target-binding peptide would allow the accumulation of a specific AMP on the surface of the target cell resulting in enhanced antimicrobial activity. Of the four peptides identified in this study, G1 was the strongest binder to the encapsulated cells of *B. anthracis*, and hence it represents the most promising peptide for use as a delivery agent that targets *B. anthracis* cells. The affinity of the G1 peptide for the vegetative cell could be increased through application of *in vitro* affinity maturation (e.g. error-prone PCR), potentially increasing its effectiveness in therapeutic applications.

The reason that the G1 peptide bound only to encapsulated cells but not the capsule extract is not clear. Our confocal microscopy result clearly demonstrated that the G1 peptide targeted specifically the encapsulated ΔAmes-1 but not the un-encapsulated Sterne 32F2. Therefore, it is unlikely that the failure of the G1 to bind the capsule extract was caused by the loss of a non-capsule compound to which the G1 might bind during extraction. As a potential delivery agent for clinical applications, the ability of a peptide to bind the encapsulated cells (rather than the capsule extract) is essential to achieve targeted killing of vegetative cells of *B. anthracis*.
